# The ALR-RSI score is a valid and reproducible scale to assess psychological readiness before returning to sport after modified Broström-Gould procedure

**DOI:** 10.1007/s00167-022-06895-7

**Published:** 2022-01-25

**Authors:** Charles Pioger, Stéphane Guillo, Pierre-Alban Bouché, François Sigonney, Marc Elkaïm, Thomas Bauer, Alexandre Hardy

**Affiliations:** 1 Department of Orthopaedic Surgery, Ambroise Paré Hospital, Paris Saclay University, 9, Avenue Charles de Gaulle, 92100 Boulogne-Billancourt, France; 2 Center for Orthopaedic Sports Surgery, Bordeaux-Mérignac, France; 3 Department of Orthopaedic Surgery, Lariboisière Hospital, Paris University, Paris, France; 4 Clinique de Tournan, Tournan-en-Brie, France; 5 Ramsay Santé, Clinique du Sport Paris V, Paris, France

**Keywords:** ALR-RSI, Ankle, Modified Broström-Gould, Psychological, Return to sport

## Abstract

**Purpose:**

Psychological readiness scores have been developed to optimize the return to play in many sports-related injuries. The purpose of this study was to statistically validate the ankle ligament reconstruction-return to sport injury (ALR-RSI) scale after modified Broström-Gould (MBG) procedure.

**Methods:**

A similar version of the ACL-RSI scale with 12 items was adapted to quantify the psychological readiness to RTS after MBG and to describe construct validity, discriminant validity, feasibility, reliability and internal consistency of the scale, according to the COSMIN methodology. The term “knee” was replaced by “ankle”. The AOFAS and Karlsson scores were used as references patient-related outcome measurements (PROMs).

**Results:**

A total of 71 patients were included. The ALR-RSI score after MBG procedure was highly (*r* > 0.5) correlated to the AOFAS and Karlsson scores, with a Pearson coefficient *r* = 0.69 [0.54–0.80] and 0.72 [0.53–0.82], respectively. The mean ALR-RSI score was significantly greater in the subgroup of 55 patients who resumed sports activity compared to those that no longer practiced sport: 61.9 (43.8–79.6) vs 43.4 (25.0–55.6), (*p* = 0.01). The test–retest showed an “excellent” reproducibility with a *ρ* intraclass correlation coefficient of 0.93 [0.86–0.96]. The Cronbach’s alpha statistic was 0.95, attesting an “excellent” internal consistency between the 12 ALR-RSI items.

**Conclusion:**

The ALR-RSI score is a valid and reproducible tool for the assessment of psychological readiness to RTS after an MBG procedure for the management of CLAI, in a young and active population. The ALR-RSI score may help to identify and counsel athletes on their ability to return to sport.

**Level of evidence:**

III.

**Supplementary Information:**

The online version contains supplementary material available at 10.1007/s00167-022-06895-7.

## Introduction

Inversion ankle trauma is one of the most frequently sports-related injury and usually involves partial or complete rupture of the anterior talofibular ligament (ATFL) [7, 12, 29]. Conservative treatment and functional rehabilitation remain the standard management for acute ankle sprains, with satisfactory outcomes [24], but controversy persists about the prevention of ankle sprain recurrence [11]. There are a still number of patients who experience ankle swelling, pain and/or a feeling of instability [25, 36]. Among them, > 20% experience chronic lateral ankle instability (CLAI), defined as recurrent acute sprain, giving way of the ankle with a perception of an insecure ankle by the patient, and avoidance/adaptation of sport activities, for at least 1 year [9, 29]. This instability can lead to cartilage damage [30], kinematic disorders [4] and early osteoarthritis of the ankle [32]. Surgery should be considered for patients with sporting demands and symptomatic ankle instability.

The modified Broström-Gould procedure (MBG) remains the gold standard for the management of CLAI and consists in the association of the ATFL repair (retention and direct suture) and the transfer of the extensor retinaculum [6, 16, 29]. The all-arthroscopic approach is increasingly used, to assess and address any associated intra-articular lesions with reduced morbidity for the patient [19, 21, 37].

In a young and active population who underwent anatomic repair surgery, one of their expectations is the ability to resume sports activity and patients are usually anxious to know the achievable level of play. Although the MBG is safe and allows most patients to resume preinjury sports activities, it has been reported that around 25% are unable to return to sport (RTS) [18, 21]. Some reports suggest that physical impairments are not sufficient to explain these low RTS rates following sport-related injuries and highlighted the role of psychological factors in the RTS process [1, 23]. The assessment of the motivation, self-confidence in performance and fear remains a key element to take into account before resuming sport activities. Therefore, psychological measurements scales have been developed to optimize the RTS rate and reduce the risk of surgical failure in many athletic injuries. In particular, Webster et al. [33] designed a scale of 12 items in patients following anterior cruciate ligament reconstruction (ACLR) to assess their psychological readiness to resume sports. This was followed by the development of numerous psychological assessment scales for return to sport after sport-related injuries, including shoulder instability (SIRSI) [8], hip arthroscopy with femoroacetabular impingement (FAI) syndrome (Hip-RSI) [35] or ankle ligament reconstruction (ALR-RSI) [28]. To date, no tool exists to analyze the psychological readiness after MBG procedure.

The main purpose of this study was to statistically validate the ankle ligament reconstruction-return to sport injury (ALR-RSI) scale using a population of patients who underwent an MBG surgery. A similar version of the ACL-RSI scale with 12 items was adapted to quantify the psychological readiness to RTS after MBG and to describe construct validity, discriminant validity, feasibility, reliability and internal consistency of the scale. Physicians and patients could use this tool to ensure psychological readiness to return to sport after MBG procedure.

## Materials and methods

### Study design

Institutional review board approval (COS-RGDS-2021-06-003) was granted for the study and all patients provided informed consent to participate.

This study identified and enrolled patients who underwent ankle ligament repair for the treatment of CLAI, in 2018 and 2019 via database of three surgical units by searching for relevant diagnostic codes. From this group, inclusion criteria comprised the following: minimum follow-up of 2 years, > 18 years old, sport activity prior to surgery and no associated lesion during the procedure. An all inside endoscopic MBG procedure was performed in all cases [10].

### ALR-RSI scale

The ALR-RSI was similar of the scale validated for the ankle ligament reconstruction and adapted from the ACL-RSI score [2]. The word “knee” was replaced by “ankle”. It contains 12 items thought to capture the psychological readiness before RTS and include: emotions (5 items), confidence in performance (5 items) and risk evaluation (2 items). The total score was equal to the sum of the 12 answers and divided by 1.2 to obtain a percentage. The score scale goes from 0 (lowest psychological readiness) to 100 (highest psychological readiness).

The last version of the ALR-RSI was validated following the international Consensus-based Standards for the selection of health status Measurement Instruments (COSMIN) guidelines [20]. An additional and standardized questionnaire was designed to capture demographic data and to collect two valid and reliable functional scores. The references PROMs used were the Karlsson score [26] and the American Orthopedic Foot and Ankle Society (AOFAS) score [15].

A total of 71 patients responded to a questionnaire including the ALR-RSI scale, the Karlsson score and the AOFAS score. Participants were also asked to give consent and to complete components relating to their return to sport. The ALR-RSI was completed twice at 15-day interval.

### Statistical analysis

To describe quantitative variables, the mean and standard deviation (SD) were used. To describe dichotomous variables, the number of events and their percentage were used. A sample size of 71 produces a two-sided 95% confidence interval with a width smaller than 0.24 when the estimate of Spearman’s rank correlation is above 0.75. To estimate the correlations between ALR-RSI, the total Karlsson score and the AOFAS, Spearman coefficients were used. If the coefficient was *r* > 0.5, the correlation was considered as “strong”, “moderate” if 0.5 < *r* < 0.3 and “weak” if 0.3 < *r* < 0.1. A Wilcoxon test was used to compare the “patient” and “control” groups to assess the discriminant validity. We also compared the patients who resumed sport and those who had abandon their sport activity. The Cronbach alpha coefficient was calculated to estimate the internal consistency and was “excellent” if *α* ≥ 0.90. The *ρ* intraclass correlation coefficient (ICCC) was used to evaluate the reliability. The reproducibility was “excellent” (*ρ* > 0.75) or “good” (0.75 < *ρ* < 0.40). The percentage of missing responses, the ceiling and floor effects allowed to evaluate the feasibility [31]. The statistical analyses were performed using the R software (version 3.5).

## Results

A total of 71 patients completed the survey and were included in the study. Each had undergone an ankle ligament repair with an all inside endoscopic MBG procedure.

Of these 71 cases, only two were professional athletes (2.8%), whereas 25 practiced sport activity in competition (35.2%), 33 as recreational and regular practice (46.5%) and 11 as occasional practice (15.5%). The main scores outcomes and the distribution of sports commonly practiced by the study population are summarized in Table 1.

**Table 1:** Baseline characteristics

Parameters	Values	*N*	Statistics
Gender	Female	35	49.3%
	Male	36	50.7%
Follow-up (years)		71	2.6 (2.0; 3.7)
ALR-RSI total		71	58.3 [41.3; 77.5]
Karlsson total		71	83.3 [74.7; 88.7]
AOFAS total		71	85.0 [74.0; 91.0]
Sport recovery	No	16	22.5%
	Yes	55	77.5%
If yes	Sport change	15	27.3%
	Same sport, inferior level	19	34.5%
	Same sport, same level	21	38.2%
Sport level	Competition	25	35.2%
	Casual leisure level	11	15.5%
	Regular leisure level	33	46.5%
	Professional	2	2.8%
Sport	Athletics	1	1.4%
	Rowing	1	1.4%
	Badminton	2	2.8%
	Basketball	6	8.5%
	Running	10	14.1%
	Dance	2	2.8%
	Climbing	1	1.4%
	Soccer	20	28.2%
	Gymnastic	2	2.8%
	Handball	6	8.5%
	Struggle	1	1.4%
	Walking	4	5.6%
	Motocross	1	1.4%
	Bodybuilding	3	4.2%
	Swimming	3	4.2%
	Rugby	6	8.5%
	Squash	2	2.8%

### Psychometric analysis

#### Convergent and structural validity (Tables 2, 3)

**Table 2:** Correlation between the ALR-RSI score and the Karlsson score

Coefficient	ALR-RSI (/100)	Karlsson total (/100)	Pain (/36)	Other symptoms (/28)	ADL (/68)	Sport (/20)	ARQL (/16)
	58.3 [41.3–77.5]	83.33 [74.7–88.7]	32 [27.0–34.0]	17 [15.0–19.0]	67 [60.5–68.0]	16 [12.5–18.5]	10 [7.0–13.0]
Spearman		0.69 [0.54–0.80]	0.54 [0.34–0.71]	0.20 [0.03–0.41]	0.47 [0.27–0.65]	0.61 [0.44–0.74]	0.82 [0.72–0.89]

**Table 3:** Correlation between the ALR-RSI score and the AOFAS score

Coefficient	ALR-RSI (/100)	AOFAS total (/100)
	58.3 [41.3–77.5]	85.0 [74.0–91.0]
Spearman		0.72 [0.53–0.82]

The ALR-RSI scale was highly (*r* > 0.5) and significantly correlated to the reference PROMs with a Pearson correlation coefficient *r* = 0.69 [0.54–0.80] regarding the Karlsson score and *r* = 0.72 [0.53–0.82] for the AOFAS. Also, the ALR-RSI scores of RTS subgroup were found discriminant. The mean ALR-RSI score was significantly higher among the 55 patients who resumed sports activity compared to those that no longer practiced sport: 61.9 (43.8–79.6) vs 43.4 (25.0–55.6), (*p* = 0.01).

#### Feasibility

No item of the ALR-RSI was missed. The floor effect, defined as the proportion of patients with the minimum score, ranged from 0 to 1.7%, and the ceiling effect relating to the highest score ranged from 4.2 to 35.2%.

#### Reliability (Fig. 1; Table 4)

**Fig. 1:**
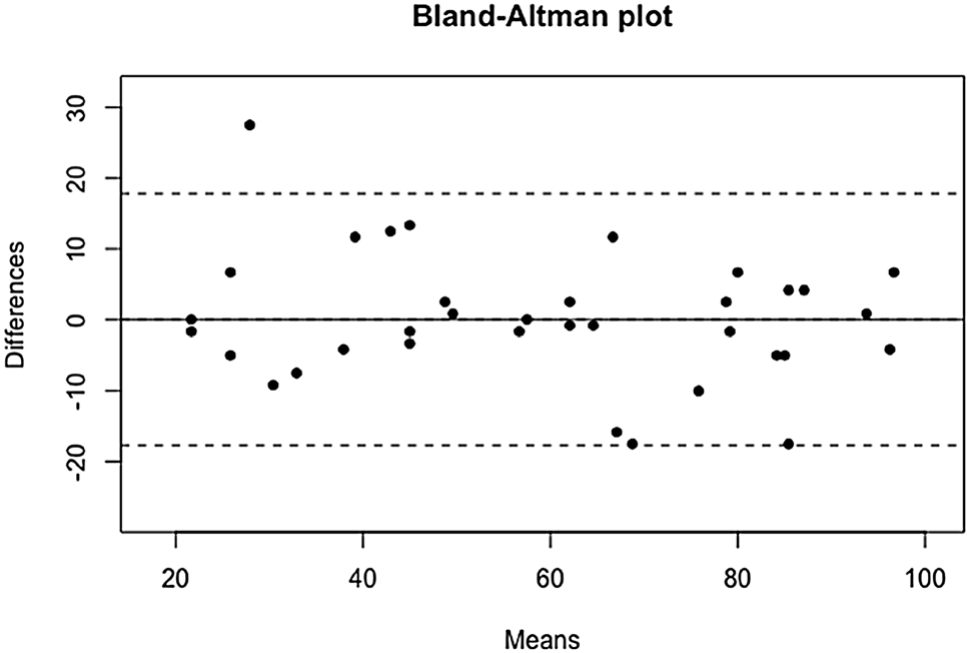
Reproducibility of the ALR-RSI score with the test–retest: Bland–Altman plot

**Table 4:** Reproducibility of the ALR-RSI with the test–retest

Coefficient	ALR-RSI 1 (/100)	ALR-RSI 2 (/100)
	58.3 [41.3–77.5]	59.2 (37.5–80.4)
ICC		*ρ* = 0.93 [0.86–0.96]

The reliability of the ALR-RSI score was explored from the calculation of the *ρ* intraclass correlation coefficient (ICCC). In the current study, the *ρ* intraclass correlation coefficient (ICCC) was found to be 0.93 [0.86–0.96], reflecting a reproducibility that was considered “excellent”. In addition, the mean ALR-RSI score was 58.3 (41.2–77.5) at the first survey completion and 59.19 (37.5–80.4) the second time.

#### Internal consistency

The Cronbach’s alpha statistic for the ALR-RSI after MBG procedure was 0.95, attesting an “excellent” internal consistency between the 12 ALR-RSI items.

#### RTS at a minimum 2-year follow-up

Fifty-five participants (77.5%) returned to sport after MBG procedure. In this group, patients resumed to the same sport with the preinjury level in 21 cases (38.2%) and at a lower performance level in 19 cases (34.5%). Sports practice was modified in 15 patients (27.3%).

## Discussion

The main finding of this study was the ALR-RSI score is a valid and reproducible tool for the assessment of psychological readiness to RTS after an MBG procedure for the management of CLAI, in a young and active population.

In the current study, 77.5% of patients returned to sport after MBG surgery, with a median follow-up of 2.6 years (2.0; 3.7). Previous literature supports this finding. In particular, a prospective study conducted by the French Arthroscopic Society observed an RTS rate around 90% following arthroscopic repair for CLAI in recreational athletes and 73% in competitive athletes. Regarding the RTS at the preinjury level, Maffulli et al. [18] presented long-term outcomes (8.7 years) about 42 athletes who underwent arthroscopic anterior talofibular Broström repair. In this cohort, the authors reported that 22 patients (58%) resumed sports at their preinjury performance level, while six changed to less demanding sports and ten patients had to abandon their sport activity. Similar outcomes were observed by Nery et al. [21] following MBG procedure at an average follow-up of 10-year follow-up. More recently, Feng et al. [6] compared outcomes between arthroscopic MBG procedure with and without repair of the ATFL remnant. No difference was found in terms of ankle function or RTS rate between the repair and non-repair group at a minimum 2-year follow-up. In particular, around 70% of patients resumed sports at their previous level in both groups. In a retrospective case series, Park et al. [22] confirmed that the absence of remnant does not affect functional outcomes. Therefore, the ALR-RSI could be used in the MBG procedure for CLAI, regardless the repair of ATFL. In a retrospective study, Lee et al. [17] focused their research on 18 elite athletes who underwent MBG operation for CLAI. The return to play (RTP) rate was 83.3% 4 months after the index surgery and 100% at 7 months. All pro athletes returned to their preinjury level. These excellent outcomes may be related to greater motivation often reported in pro athletes [3] but also to better access to professional monitoring and high-level rehabilitation. However, this report must be interpreted with cautious given the small number of patients in the cohort (*n* = 18).

The RTP timeline and the ability to resume sport at the preinjury level are primarily of concern for young and sportive population. White et al. [34] reported a lack of documented data to guide athletes in their RTP timeline. This is why, authors of further studies about surgical management of CLAI have to endeavor to track patients’ progression during rehabilitation and report complete data about RTP, including psychological readiness. To date, recommendation for RTS criteria mainly includes clinical indicators such as joint stability, muscle strength and full ROM [29]. In a recent systematic review, Hunt et al. [13] highlighted the heterogeneity and the deficiency of consistent metrics for RTS in the included studies. The authors call for standardized, valid and reproducible tools for reporting RTS. In the same way, Clanton et al. [5] pointed out the need for subjective data to determine the ability to resume sports. To this end, the ALR-RSI scale should be used in routine, because functional testing coupled with psychological assessment allows taking RTS decisions safely. The ACL-RSI score remains an example of the interest of a psychological RTS evaluation after surgery. A strong and significant correlation between this psychological scale and return to sport has been demonstrated [27].

The choice of a survey with numeric answers allows simplifying the collect of data, compared to open questionnaire. Sports surgeons and physicians can easily refer to this questionnaire to counsel patients on RTS. However, Webster and Feller examined the responsiveness of the ACL-RSI score and found a moderate responsiveness over 6 months, using anchor-based methods. Specifically, the authors showed that the ACL-RSI scale had a sufficient responsiveness to detect clinical relevant changes at a group level and was more limited at an individual level.

There are several limitations in the current study. First, a possible selection bias may have been introduced, related to the inherent nature of a retrospective study. Second, the ALR-RSI scale was initially based on a modification of the ACL-RSI score and not developed for CLAI. However, the ALR-RSI has recently been validated for ankle instability after anatomic ligament reconstruction [28]. Moreover, many psychological assessment scores after sport-related injuries have been based on the ACL-RSI scale, which has been shown to be easily transferable to other joints and pathologies [8, 14, 35].

This study validated the ALR-RSI score as a routine practice tool to assess the psychological readiness to RTS after Broström procedure in patients with CLAI.

## Conclusion

Through the results of this cohort, the ALR-RSI has to be considered as RTS metric tool to provide a clear message to patients who underwent MBG procedure for CLAI. Orthopaedic surgeons may use these findings to counsel and set expectations with their active patients, using evidence-based medicine on their ability to return to their favorite sport.

## Supplementary Information

Below is the link to the electronic supplementary material.

Supplementary file1 (DOCX 18 kb)
