# Correction to: The ALR-RSI score is a valid and reproducible scale to assess psychological readiness before returning to sport after modified Broström-Gould procedure

**DOI:** 10.1007/s00167-022-06917-4

**Published:** 2022-02-22

**Authors:** Charles Pioger, Stéphane Guillo, Pierre-Alban Bouché, François Sigonney, Marc Elkaïm, Thomas Bauer, Alexandre Hardy

**Affiliations:** 1 Department of Orthopaedic Surgery, Ambroise Paré Hospital, Paris Saclay University, 9, Avenue Charles de Gaulle, 92100 Boulogne-Billancourt, France; 2 Center for Orthopaedic Sports Surgery, Bordeaux-Mérignac, France; 3 Department of Orthopaedic Surgery, Lariboisière Hospital, Paris University, Paris, France; 4 Clinique de Tournan, Tournan-en-Brie, France; 5 Ramsay Santé, Clinique du Sport Paris V, Paris, France

## Correction to: Knee Surgery, Sports Traumatology, Arthroscopy 10.1007/s00167-022-06895-7

The article “The ALR‑RSI score is a valid and reproducible scale to assess psychological readiness before returning to sport after modified Broström‑Gould procedure”, written by Charles Pioger, Stéphane Guillo, Pierre‑Alban Bouché, François Sigonney, Marc Elkaïm, Thomas Bauer and Alexandre Hardy was originally published electronically on the publisher’s internet portal on 25 January 2022 without open access. With the author(s)’ decision to opt for Open Choice the copyright of the article changed on 18 February 2022 to © The Author(s) 2022 and the article is forthwith distributed under a Creative Commons Attribution 4.0 International License, which permits use, sharing, adaptation, distribution and reproduction in any medium or format, as long as you give appropriate credit to the original author(s) and the source, provide a link to the Creative Commons licence, and indicate if changes were made. The images or other third party material in this article are included in the article’s Creative Commons licence, unless indicated otherwise in a credit line to the material. If material is not included in the article’s Creative Commons licence and your intended use is not permitted by statutory regulation or exceeds the permitted use, you will need to obtain permission directly from the copyright holder. To view a copy of this licence, visit http://creativecommons.org/licenses/by/4.0/

The original article has been corrected.

