# Supplementary material for: The ALR-RSI score is a valid and reproducible scale to assess psychological readiness before returning to sport after modified Broström-Gould procedure
Source: Knee Surg Sports Traumatol Arthrosc. 2022 Jan 25;30(7):2470–5. doi: 10.1007/s00167-022-06895-7 (PMC9206630; doi:10.1007/s00167-022-06895-7)
Supplement: Supplementary file 1 — Supplementary file1 (DOCX 18 kb) [file 167_2022_6895_MOESM1_ESM.docx]

**Appendix:** ALR-RSI scale

ALR-RSI scale

Please answer the following questions referring to your main sport prior to injury. For each question, tick a box □✓ between the two descriptions to indicate how you feel right now relative to the two extremes.

**1.Are you confident that you can perform at your previous level of sport participation?**

| Not at all confident | □ | □ | □ | □ | □ | □ | □ | □ | □ | □ | □ | Fully confident |
| --- | --- | --- | --- | --- | --- | --- | --- | --- | --- | --- | --- | --- |
|  | 0 | 1 | 2 | 3 | 4 | 5 | 6 | 7 | 8 | 9 | 10 |  |

**2.Do you think you are likely to re-injure your ankle by participating in your sport?**

| Extremely likely | □ | □ | □ | □ | □ | □ | □ | □ | □ | □ | □ | Not likely at all |
| --- | --- | --- | --- | --- | --- | --- | --- | --- | --- | --- | --- | --- |
|  | 0 | 1 | 2 | 3 | 4 | 5 | 6 | 7 | 8 | 9 | 10 |  |

**3.Are you nervous about playing your sport?**

| Extremely nervous | □ | □ | □ | □ | □ | □ | □ | □ | □ | □ | □ | Not nervous at all |
| --- | --- | --- | --- | --- | --- | --- | --- | --- | --- | --- | --- | --- |
|  | 0 | 1 | 2 | 3 | 4 | 5 | 6 | 7 | 8 | 9 | 10 |  |

**4.Are you confident that your ankle will not give way by playing your sport?**

| Not at all confident | □ | □ | □ | □ | □ | □ | □ | □ | □ | □ | □ | Fully confident |
| --- | --- | --- | --- | --- | --- | --- | --- | --- | --- | --- | --- | --- |
|  | 0 | 1 | 2 | 3 | 4 | 5 | 6 | 7 | 8 | 9 | 10 |  |

**5.Are you confident that you could play your sport without concern for your ankle?**

| Not at all confident | □ | □ | □ | □ | □ | □ | □ | □ | □ | □ | □ | Fully confident |
| --- | --- | --- | --- | --- | --- | --- | --- | --- | --- | --- | --- | --- |
|  | 0 | 1 | 2 | 3 | 4 | 5 | 6 | 7 | 8 | 9 | 10 |  |

**6.Do you find it frustrating to have to consider your ankle with respect to your sport?**

| Extremely frustrating | □ | □ | □ | □ | □ | □ | □ | □ | □ | □ | □ | Not at all frustrating |
| --- | --- | --- | --- | --- | --- | --- | --- | --- | --- | --- | --- | --- |
|  | 0 | 1 | 2 | 3 | 4 | 5 | 6 | 7 | 8 | 9 | 10 |  |

**7.Are you fearful of re-injuring your ankle by playing your sport?**

| Extremely fearful | □ | □ | □ | □ | □ | □ | □ | □ | □ | □ | □ | No fear at all |
| --- | --- | --- | --- | --- | --- | --- | --- | --- | --- | --- | --- | --- |
|  | 0 | 1 | 2 | 3 | 4 | 5 | 6 | 7 | 8 | 9 | 10 |  |

**8.Are you confident about your ankle holding up under pressure?**

| Not at all confident | □ | □ | □ | □ | □ | □ | □ | □ | □ | □ | □ | Fully confident |
| --- | --- | --- | --- | --- | --- | --- | --- | --- | --- | --- | --- | --- |
|  | 0 | 1 | 2 | 3 | 4 | 5 | 6 | 7 | 8 | 9 | 10 |  |

**9.Are you afraid of accidentally injuring your ankle by playing your sport?**

| Extremely afraid | □ | □ | □ | □ | □ | □ | □ | □ | □ | □ | □ | Not at all afraid |
| --- | --- | --- | --- | --- | --- | --- | --- | --- | --- | --- | --- | --- |
|  | 0 | 1 | 2 | 3 | 4 | 5 | 6 | 7 | 8 | 9 | 10 |  |

**10. Do thoughts of having to go through surgery and rehabilitation prevent you from playing your sport?**

| All of the time | □ | □ | □ | □ | □ | □ | □ | □ | □ | □ | □ | None of the time |
| --- | --- | --- | --- | --- | --- | --- | --- | --- | --- | --- | --- | --- |
|  | 0 | 1 | 2 | 3 | 4 | 5 | 6 | 7 | 8 | 9 | 10 |  |

**11. Are you confident about your ability to perform well at your sport?**

| Not at all confident | □ | □ | □ | □ | □ | □ | □ | □ | □ | □ | □ | Fully confident |
| --- | --- | --- | --- | --- | --- | --- | --- | --- | --- | --- | --- | --- |
|  | 0 | 1 | 2 | 3 | 4 | 5 | 6 | 7 | 8 | 9 | 10 |  |

**12. Do you feel relaxed about playing your sport?**

| Not at all relaxed | □ | □ | □ | □ | □ | □ | □ | □ | □ | □ | □ | Fully relaxed |
| --- | --- | --- | --- | --- | --- | --- | --- | --- | --- | --- | --- | --- |
|  | 0 | 1 | 2 | 3 | 4 | 5 | 6 | 7 | 8 | 9 | 10 |  |

**ALR-RSI score total x 100 / 120 = __ %**
